# Assessment of Knowledge of Learning Disabilities and Workshop for Palestinian Primary School Teachers: A Single-Center Study

**DOI:** 10.1192/bjo.2025.10288

**Published:** 2025-06-20

**Authors:** Nizar Marzouqa, George Ibrahim, Nora Marzouqa, Alessia Grazia Morea

**Affiliations:** 1 Bethlehem Psychiatric Hospital, Bethlehem, Palestine; 2 Jemima, Beit Jala, Palestine; 3 Oxford University, Oxford, United Kingdom; 4 Pro Terra Sancta, Bethlehem, Palestine

## Abstract

**Aims:** To assess primary school teachers’ knowledge of learning disabilities, and design interactive lectures targeting revealed gaps.

**Methods:** A 30-question survey was designed based on previously published research alongside a discussion with teacher supervisors. It was comprised of true-or-false or multiple-choice questions, divided into five parts: demographic details, learning disabilities general knowledge, specific learning disabilities, developmental disorders, and management.

A Four-session workshop was held at Terra Santa College in Bethlehem from December 2024 to January 2025, provided by a speech therapist and a psychiatry resident, with the support of Pro Terra Sancta (Bethlehem). Each session was two hours long providing knowledge and interactive activities followed by discussion. The sessions correlated with the different parts of the questionnaire. The first session focused on normal child development, learning disability types, and manifestations. The second session covered specific learning disorders: dyslexia, dysgraphia, and dyscalculia. The third session had an emphasis on developmental disorders: autism spectrum disorder and Attention-Deficit/Hyperactivity Disorder. Finally, the fourth session was about multidisciplinary management, the role of teachers, and individualized learning plans.

**Results:** Twenty-eight Palestinian primary school teachers filled out the pre-assessment survey. They were all female, of different age groups (9 teachers under 35 years old, 12 between 36–45, and 7 older than 45), and several disciplines (including 9 teaching Languages (32.1%)). Among the participants, 57.1% had related training previously, 92.9% said they teach a student with a learning disability, and 75% described teaching these children as ‘very challenging’ (n=4) or ‘challenging’ (n=17). The teachers’ average overall score on the pre-assessment was 55% correct, with the highest section mean being developmental disorders (62%), compared with specific learning disorders (34%). Interestingly, most mistakes were related to the misconception that learning or development disorders can be diagnosed primarily with brain imaging. A quick analysis, using RStudio 2024.04, showed a significant difference (p<0.05) in overall scores when comparing those who had prior similar training (mean=60.6%) and those who had not (mean=51.5%), with no significant differences among other demographic measures.

The feedback was overwhelmingly positive, with teachers reporting more confidence and enthusiasm to interact with students with learning and developmental disabilities. Specifically, they cited the activities as interactive and relevant to their experiences.

**Conclusion:** Teachers have a big role in identifying and supporting children with learning disabilities, therefore, awareness campaigns should target this population. Such small studies can lay the groundwork for future research and workshops.

